# Partners in root nodule symbiosis respond uniquely to heavy metal stresses in a host genotype-dependent manner

**DOI:** 10.1038/s41598-025-17827-z

**Published:** 2025-09-29

**Authors:** Sanhita Chakraborty, Reena Sharma, Aditi Bhat, Shaun J. Curtin, Jiangqi Wen, Kirankumar S. Mysore, Timothy Paape

**Affiliations:** 1 Institute for Advancing Health Through Agriculture (IHA), Texas A&M University, College Station, TX USA; 2 Brookhaven National Laboratory, Upton, NY USA; 3 Department of Agriculture (USDA), Plant Science Research Unit, United States, St Paul, MN 55108 USA; 4 Department of Agronomy and Plant Genetics, University of Minnesota, St. Paul, MN 55108 USA; 5 Center for Plant Precision Genomics, University of Minnesota, St. Paul, MN 55108 USA; 6 Center for Genome Engineering, University of Minnesota, St. Paul, MN 55108 USA; 7 Department of Plant and Soil Sciences, Oklahoma State University, Stillwater, OK USA; 8 Department of Biochemistry and Molecular Biology, Oklahoma State University, Stillwater, OK USA; 9 Department of Soil and Crop Sciences, Texas A&M University, College Station, TX USA; 10 USDA-ARS Responsive Agriculture Food Systems Research Unit, College Station, TX USA

**Keywords:** Heavy metal, Nodule, Symbiosis, ABC transporter, Dual transcriptomics, ICP-MS, Plant sciences, Plant stress responses, Abiotic

## Abstract

The mutualistic symbiosis between legume roots and soil rhizobia culminates in the formation of root nodules, where nitrogen is fixed. Root nodule symbiosis is inhibited by heavy metal stress. In this study, we investigated the relative responses of the symbiotic partners to a non-essential heavy metal cadmium (Cd) and an essential heavy metal zinc (Zn) stress and identified patterns in gene expression. We performed dual transcriptomics in nodules, using the *Medicago truncatula-Sinorhizobium meliloti* symbiotic system. Phenotypes were measured in the wild-type *Medicago truncatula* and a mutant in an *ABC* transporter gene (*Mtabcg36*), which showed compromised nodule formation in control conditions and further after heavy metal treatment. We observed that the rhizobia were particularly sensitive to Zn in mutant nodules. The greatest degree of differential gene expression in the host plant were observed under Cd and Zn treatments in wild-type nodules. Most Cd-regulated host genes were also differentially regulated by Zn, revealing little discernment between an essential and a non-essential ion under increased exposure. Furthermore, the host response to both the stresses affected auxin and iron homeostasis genes in a host genotype-dependent manner. Our results suggested impaired cadmium export from the mutant nodules. These results have potential implications in agricultural management systems and bioremediation strategies.

**Supplementary Information:**

The online version contains supplementary material available at 10.1038/s41598-025-17827-z.

## Introduction

The ionic composition of the rhizosphere is important for plant growth and development. Heavy metals such as manganese, iron, copper, and zinc (Zn) act as plant micronutrients at low concentrations but can be phytotoxic at higher concentrations. On the other hand, no function has been ascribed to heavy metals such as lead, cadmium (Cd), or mercury, and these are considered phytotoxic even at low concentrations. Geological processes such as weathering of rocks as well as anthropogenic activities such as accumulation of agrochemical and industrial waste can cause high levels of heavy metals in arable lands. These metal ions are readily taken up by the roots and often transported to the shoots, where they can enter the food web via edible plant parts such as seeds and leaves^1,2^. Excess levels of heavy metals can disrupt ionic homeostasis in cells and cause oxidative stress, affecting various processes in plants, including germination, photosynthesis, nutrient uptake, and their interactions with beneficial soil microorganisms^3–6^.

Legumes develop symbiotic relationships with nitrogen-fixing bacteria (rhizobia), culminating in symbiotic root organs called nodules, where atmospheric nitrogen is converted into a form usable by plants. Nodule formation is extremely sensitive to the abiotic environment^7,8^. In the model legume, *Medicago truncatula*, heavy metal stress (Cd, Hg, Zn) reduces the number of nodules^9,10^and inhibits root growth variably among genotypes^11,12^. In legumes, heavy metals also lower the accumulation of free amino acids, total protein and nitrogen content, and decrease the activity of nitrogenase, the bacterial enzyme that catalyzes the breakdown of atmospheric nitrogen^13^. Both ion and hormone homeostasis are essential for successful nodulation and maintaining symbiosis. Several metal ion transporter-encoding and hormone-responsive genes have been implicated in nodulation^7^.

ATP-Binding Cassette (ABC) transporter proteins are found in all forms of life. ABC transporters are membrane-bound proteins that use the energy generated from ATP hydrolysis to transport substrates across membranes. Their nucleotide-binding domains are conserved, while the transmembrane substrate-binding domains are diverse, often due to substrate specificity. ABC proteins in plants transport a variety of substrates such as hormones, pigment precursors, lipid precursors, and metal ions. In the model legume *Medicago truncatula*, the *ABC* genes have diversified into several clades, and some are known to be involved in nodule formation, including one in the G-clade, *MtABCG36*^14^–^20^. *MtABCG36* shows variable expression patterns in various tissues during symbioses and is significantly induced during mycorrhizal symbiosis (Medicago Gene Expression Atlas)^15^. In *Arabidopsis thaliana* and rice, the expression of *ABCG36* orthologs in both species was induced by Cd stress in roots more strongly than in leaves, and knockdown and knockout mutations resulted in decreased root growth and greater sensitivity to increased Cd concentrations^21,22^.

Dual transcriptomics is a powerful tool for simultaneously studying the responses of endosymbiotic species and their hosts in response to internal or external cues^23–28^. Here, we studied the effects of Cd and Zn stress on the *Medicago truncatula-Sinorhizobium meliloti* symbiotic partnership in the nodules of two host genotypes using dual transcriptomics. Our goals were to: (1) study the relative responses of the symbiotic partners to these stresses, (2) compare gene expressions in response to an essential and a non-essential heavy metal, and (3) assess the contribution of the host genotype in mediating heavy metal stress responses in nodules^18^. Because the plant host directly encountered the heavy metal treatments, we hypothesized that the host would show a stronger response than the microsymbiont. Furthermore, given the differences in the known nutritional roles between the two metals, we hypothesized that exogenous Cd would have a greater impact on the nodule transcriptome, and that there will be moderate overlap in differentially expressed genes (DEGs) in response to these treatments.

## Results

### Heavy metal treatments and the host genotype influence plant biomass and symbiosis with rhizobium

**Fig. 1:**
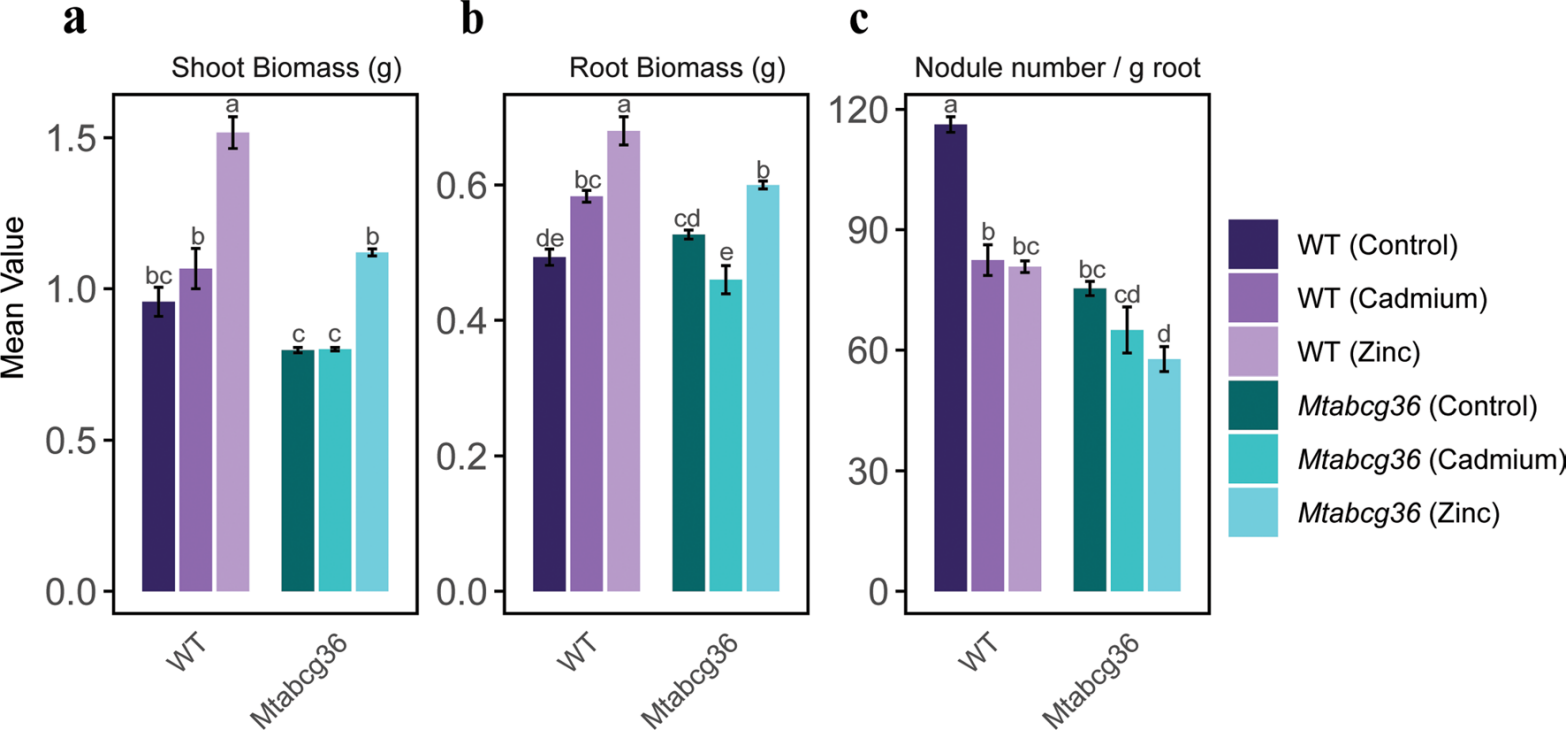
Quantification of shoot and root biomass, and normalized nodule number. Biomass measures for (**a**) shoot, (**b**) root and (**c**) nodule number per gram of root in wild-type (WT) and *Mtabcg36* mutant plants that were inoculated with *Sinorhizobium meliloti Sm2011* and treated with cadmium, zinc, or untreated (control). One week after the treatment, the shoot, root, and nodules were harvested. Biomass measurements used fresh weight. Five plants were tested in each condition, and the experiment repeated three times. The number of nodules indicates total nodules pooled from five plants. Error bars indicate standard error of the mean (SEM). Letters display the Tukey’s Honestly Significant Difference (HSD) test for multiple comparisons at α = 0.05. When common letters are shown above any bar, they are not significantly different.

To test the effect of heavy metal stress on nodules in the wild-type (WT) and mutant host genotypes, we subjected rhizobium-inoculated plants to Cd and Zn treatments. The control treatment contained necessary macro- and micronutrients, including a basal level of Zn (see methods for details). In the WT plants, the Zn stress resulted in increased shoot and root biomass while Cd increased root biomass only **(**Fig. 1a, b**)**. Nodule number per gram of root decreased in response to both treatments in the WT. Nodule number per gram of root was also lower in the *Mtabcg36* mutant background compared to the WT without any heavy metal treatment **(**Fig. 1c**)**, as previously observed by Curtin et al., (2017). In the *Mtabcg36* mutant genotype, Cd treatment decreased root biomass compared to control treatment, but Zn treatment increased both shoot and root biomass compared to control treatment. Furthermore, in the *Mtabcg36* mutant, Zn, but not Cd-treated roots had fewer nodules per gram of root **(**Fig. 1, S1a-c**)**. No difference was observed in nodule biomass either between untreated genotypes or treatments within each genotype, however, nodule biomass in Zn-treated mutant nodules was significantly lower than Zn-treated wild-type nodules (Supplementary Fig. S2**)**. These results show that both the heavy metal treatments as well as the plant genotype influenced the plant growth and nodule number, and both Cd and Zn inhibited nodule formation.

### Heavy metal treatments have large effects on the host but not the microsymbiont transcriptome in the nodule

**Fig. 2:**
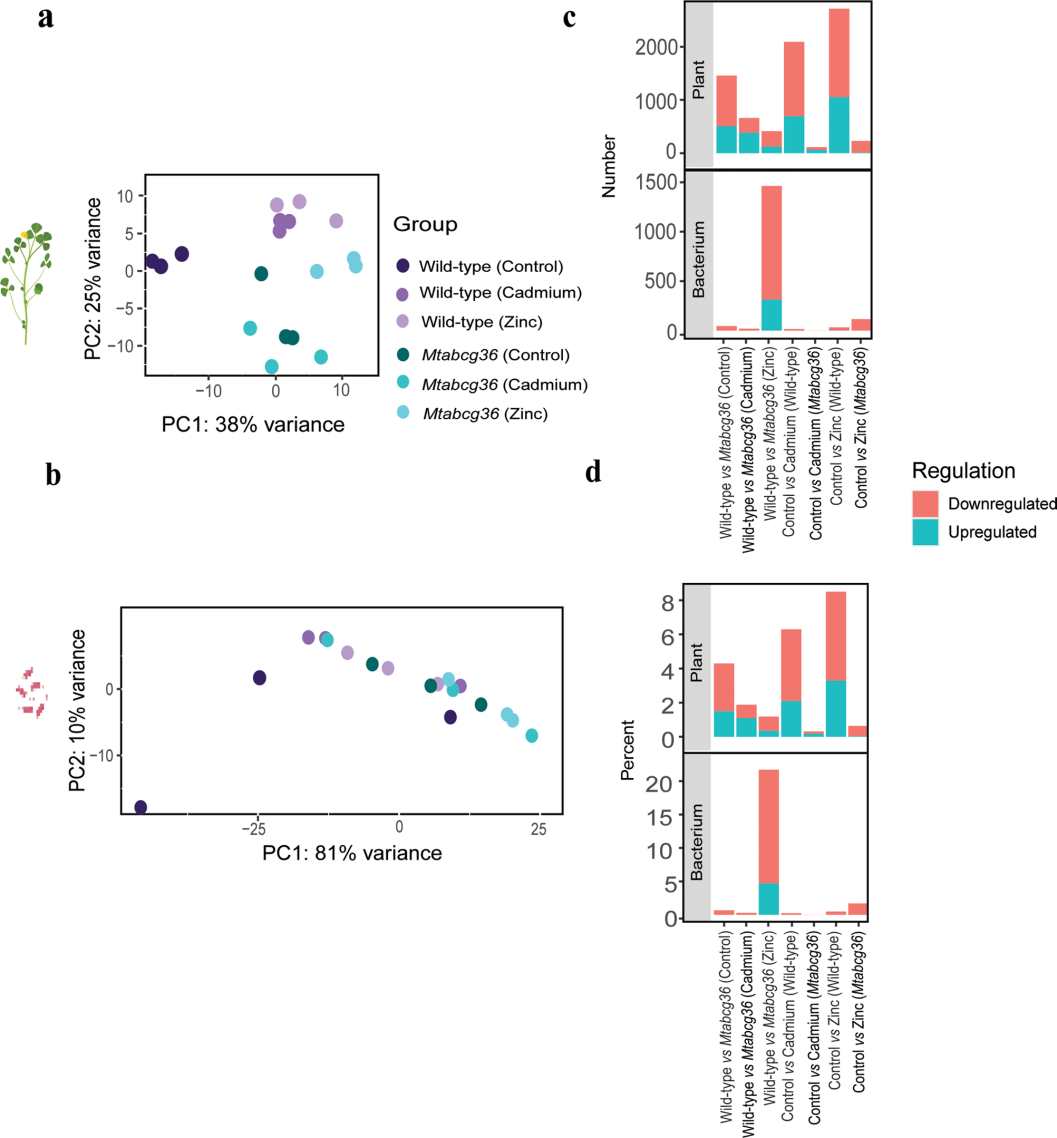
Mutation in *MtABCG36* and heavy metal treatments strongly influence the host transcriptome in the nodule. Principal component analysis (PCA) of the (**a**) plant and (**b**) bacterial transcriptome in the wild-type or *Mtabcg36* mutant nodules subjected to cadmium or zinc treatments. Control, cadmium or zinc treated samples were colored using purple hues for wild-type and green hues for mutant. Stacked bar plots showing the number (**c**) or percent (**d**) of differentially expressed genes (DEGs) using adjusted *p*-value < 0.05 (False Discovery Rate, FDR) for comparisons between control and Cd or Zn treated plants, and comparisons between WT and mutant. Red indicates downregulated DEGs and green indicates upregulated DEGs.

To quantify the transcriptional responses in the two plant genotypes resulting from Cd and Zn stress in the context of rhizobium-legume symbiosis, we performed dual RNA-seq from the nodules to produce genome-wide expression profiles for both symbiotic partners from the WT and *Mtabcg36* mutant backgrounds **(**Supplementary Fig. S3**)**. In the plant, principal component analysis (PCA) revealed distinct clustering of gene expression by plant genotype, and by stress treatments **(**Fig. 2a**)**. On the bacterial side, however, no clear clustering of gene expression was observed **(**Fig. 2b**).**

We questioned whether the absence of clustering on the bacterial side was a technical artifact, such as sequencing coverage, or had any potential biological explanation. We therefore compared the total mapped read counts in each condition for each biological replicate for the bacterium and the plant in the dual RNA-seq data. The plant genome has about five times the number of annotated genes than in the bacterium **(Supplementary Fig. S4a)** and about twice (on average across all samples) the number of mapped reads in the plant compared with the bacteria **(**Fig. S4b**)**. When mapped reads were normalized to the total number of genes in either species, both the host plant and the bacteria showed comparable coverage **(**Supplementary Fig. S4c, d**)**. These results corroborated the fact that the lack of clustering of gene expression on the bacterial side had a biological relevance.

To gain a deeper insight into the effect of each treatment, host genotype, and their interactions, on the dual transcriptome, we conducted pairwise differential expression analyses of all biologically meaningful combinations of conditions **(**Supplementary Tables S1, S2**)**. Our analyses revealed that all treatments, genotypes, and their interactions influenced the host transcriptome to different degrees. The Cd and Zn treatments in the WT nodules resulted in the largest number of DEGs in the host plant. These treatments led to 2087 (2.1% of the total number of genes) and 2711 (3.3% of the total number of genes) plant DEGs in WT nodules, respectively (*p-*adj < 0.05) **(**Fig. 2c, d**)**. In contrast, the mutant genotype showed dramatically small numbers of DEGs in response to Cd or Zn (113 (0.31%) and 229 (0.64%), respectively), indicating that the effect of these metals on the plant transcriptome in the nodule is largely dependent on the plant genotype. When comparing sets of genes from the post-treatment conditions in WT vs. mutant, the number of DEGs from Cd-treated plants was greater than for Zn-treated plants. This finding was supported by the PCA which showed a greater distance between the WT Cd-treated plants and mutant Cd-treated plants on the PC2 axis compared with the separation between Zn-treated WT and Zn-treated mutant plants on the PC2 axis **(**Fig. 2a**)**.

The DEGs observed in *S. meliloti* from nodules were remarkably fewer compared with the host plant **(**Fig. 2c, d**)**. The Zn-treated WT vs. Zn-treated mutant nodules was the only category that had any considerable differential expression on the rhizobial side. Because a strong response was not observed in the rhizobia induced by Zn treatment in the WT host-plant background, or between rhizobia in the WT and mutant host-plant genotypes without any treatment, or in rhizobia in the mutant host in response to Zn (i.e., control vs. treatment), the response when comparing rhizobial gene expression after Zn treatment in the WT and the mutant host-plant backgrounds, appears to be synergistic, rather than additive **(**Supplementary Fig. S5**)**^29^. This category showed over 20% of the rhizobial genome to be differentially expressed after Zn treatment in the WT vs. mutant backgrounds. While the clustering of the RNA-seq data in the PCA from the rhizobial samples was much less obvious compared to the host plant, the Zn-treated rhizobial samples in the WT vs. mutant was among the broadest separation on the PC1 axis **(**Fig. 2b, Supplementary Fig. S6**)**. Together, these results indicate that the host and the microsymbiont behave quite differently in their response to heavy metal stresses in the nodule. At the whole transcriptome level, the host cells in the nodules appear much more sensitive to the heavy metal stresses that lead to changes in gene expression, compared to the microsymbiont.

### Several transcriptional regulator-, transporter-encoding-, and symbiotic rhizobial genes are downregulated in the *Mtabcg36* mutant nodules under Zn treatment compared to Zn-treated WT nodules

**Fig. 3:**
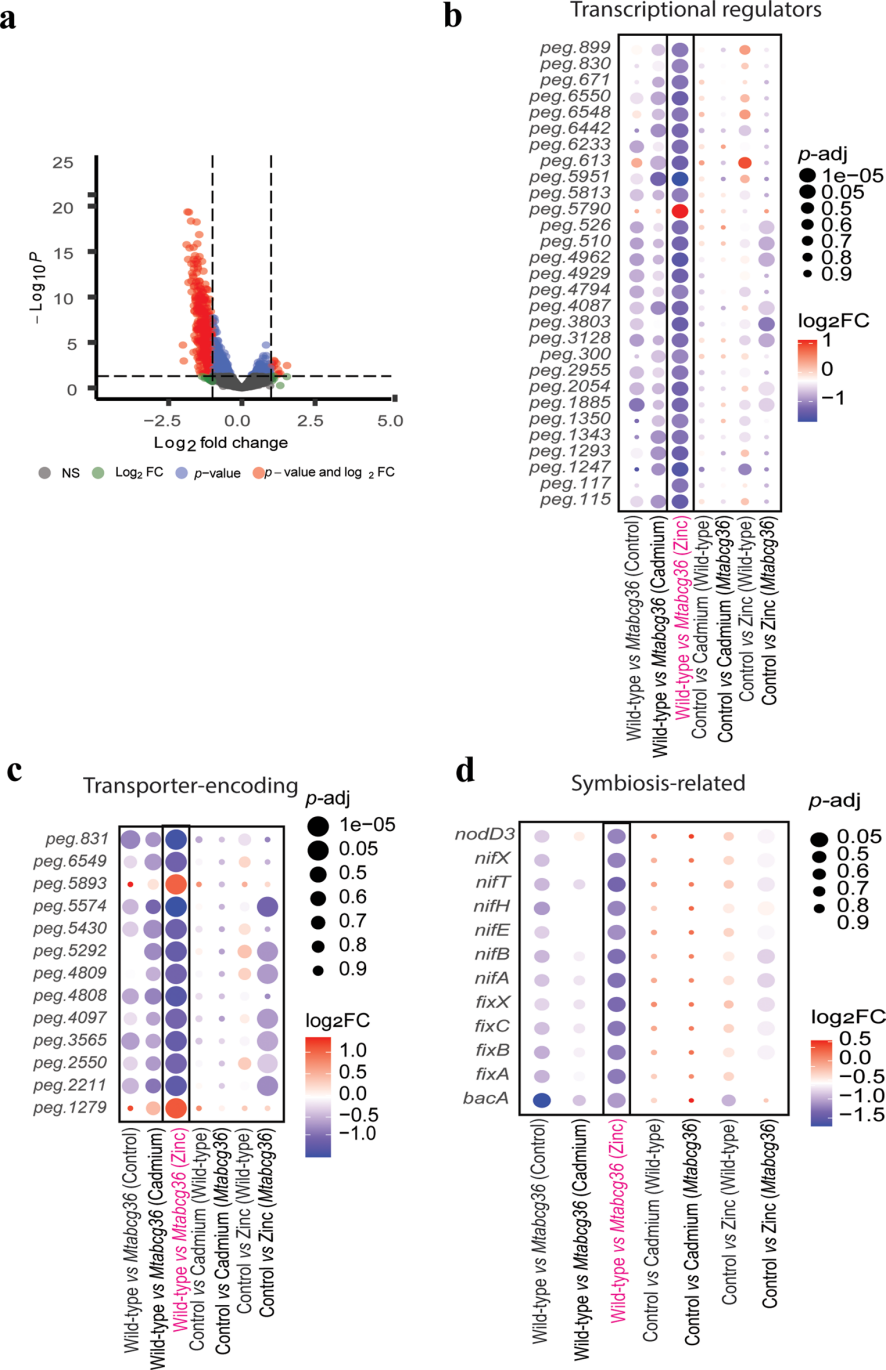
Differentially expressed transcriptional regulator-, transporter-encoding and symbiotic genes in rhizobium in the nodule in the wild-type (WT) vs. *Mtabcg36* mutant comparison under zinc (Zn) treatment. (**a**) Volcano plot showing the significant rhizobial DEGs comparing Zn treated WT vs. *Mtabcg36* mutant samples. Blue points adjusted *p*-value < 0.05, red points are |log_2_FC| > 1 and adjusted *p*-value < 0.05, gray points are non-significant, (NS). Negative log_2_FC indicates lower expression in the mutant than the WT after the Zn treatment. (b-d) Dot plots representing differential expressions of (**b**) transcriptional regulator-, (**c**) transporter-encoding, and (**d**) symbiotic rhizobial genes. Differential expression was defined as log_2_FC| > 1; *p*-adj < 0.05. The box includes only significant DEGs for the Zn-treated WT vs. *Mtabcg36* comparison. These genes may or may not be differentially regulated in any other combination of genotypes and treatments. The color of the dots represents the directionality of gene expression. The size of the dots represents the reverse of the adjusted *p*-value; larger dots represent a smaller adjusted *p*-value. Labels on y-axes of b-d are gene IDs from the RAST annotation (Tables S4-5) or known symbiosis homologs (Table S3).

The combination of Zn treatment and the host mutant background had a synergistic effect on the rhizobial transcriptome in the nodule, as neither Zn treatment nor the host mutant background alone, impacted gene expression in the rhizobia substantially. Together, they had a large effect on the rhizobial transcriptome after Zn treatment in WT and *Mtabcg36* hosts that is much greater (based on numbers of DEGs) than the added effects of control vs. Zn treatment in either the WT or mutant **(**Fig. 2C, D**).** Because Zn-treated WT vs. mutant nodules was the only comparison in *S. meliloti* that showed considerable differential gene expression at the whole transcriptome level **(**Fig. 2c, d**)**, we explored the DEGs in this category. Using a stringent threshold of |log_2_FC| >1 and *p-*adj < 0.05, we observed that the majority of DEGs were downregulated in the mutant nodules vs. WT after both were subjected to Zn treatment, suggesting that these genes were negatively regulated by Zn in the rhizobia in the *Mtabcg36* host plant nodules **(**Fig. 3a, Supplementary Table S3**)**. This category of DEGs included several genes encoding putative transcriptional regulators and transporters **(**Fig. 3b, c, Supplementary Tables S4,S5**)**. Over half of the DEGs encoding transporters were putative ABC transporters **(**Fig. 3c**)**. The Zn-treated WT vs. mutant category also included several symbiotic genes, including *nodD3*,* bacA*, and multiple *nif* and *fix* genes, which all had significantly lower expression in mutant vs. WT host background under Zn treatment **(**Fig. 3d**)**. None of the rhizobial genes in these three groups was significantly regulated by Zn in the WT host plant **(**Fig. 3b-d**)**. Combined, these findings show that host-plant genotype influences gene expression after Zn stress in the microsymbiont in the nodule, and unlike the response in the host-plant, the expression differences in the microsymbiont are much larger after Zn treatment than after Cd treatment.

### Cd and Zn act through common host genotype-dependent mechanisms to influence the host transcriptome in the nodule

**Fig. 4:**
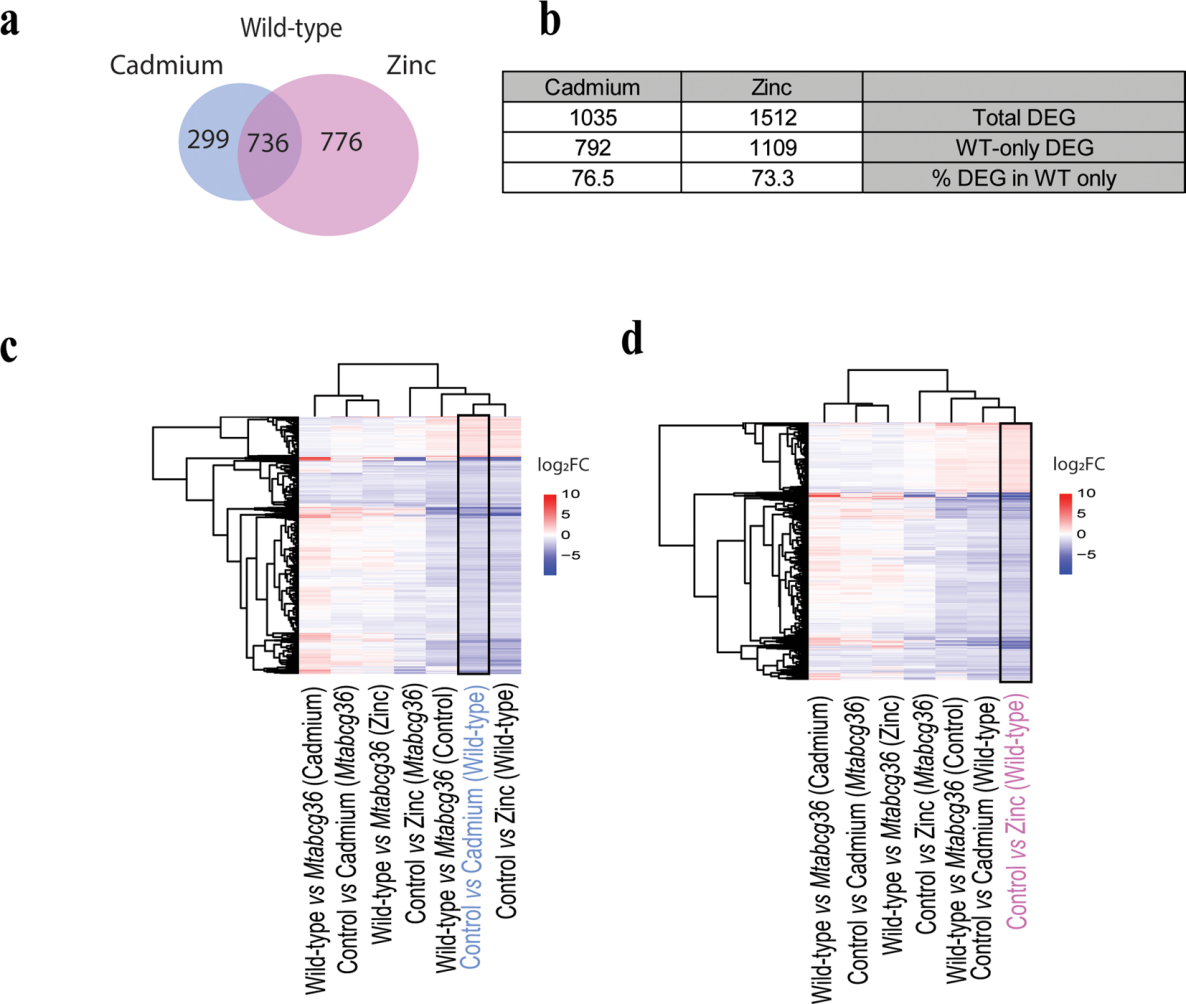
Differentially expressed host plant genes in the nodule in response to Cd and Zn. (**a**) Venn diagram showing the DEGs that were unique to either cadmium or zinc treatments, and those that overlap in wild-type R108 nodules. These genes may or may not be differentially regulated in the mutant by these treatments. (**b**) Table summarizing the total number of plant DEGs and the number and percentage of DEGs that were differentially regulated in the WT only, in response to Cd or Zn. “Total DEG” indicates DEGs in response to Cd or Zn in the WT that are, or are not differentially regulated in the mutant. “WT-only DEG” indicates DEGs in response to Cd or Zn in the WT but not the mutant, and “% DEG in WT only” indicates % DEGs in response to Cd or Zn in the WT but not the mutant (**c**) Heatmap of DEGs observed under cadmium treatment in the wild-type nodules (vertical rectangle box). (**d**) Heatmap of DEGs observed under zinc treatment in the wild-type nodules (vertical rectangle box). The colors represent the directionality of gene expression. For the heatmaps, the genes may or may not be differentially regulated in the other combinations of genotypes and treatments other than the ones specified in blue/pink X-axis label and enclosed in the vertical box. DEGs are defined as |log_2_FC| > 1; *p*-adj < 0.05. Clustering was using expression level change (y-axis) and sample/treatment (x-axis) on the heatmaps.

Unlike the rhizobial symbiont, the host plant showed far greater transcriptional responses in all comparisons **(**Fig. 2c, d**)**. In the WT, Cd and Zn treatments resulted in the largest number of DEGs (|log_2_FC| >1, *p-*adj < 0.05) showing over 6% (of the total number of plant genes) and 8% (of the total number of plant genes) DEGs that responded to the Cd and Zn treatments, respectively **(**Fig. 2C, D**).** Together, these treatments resulted in a total of 1,811 DEGs in the WT, which may or may not be differentially regulated in the mutant **(**Fig. 4a**)**. Over 70% of these genes were not differentially regulated in the mutant, corroborating the role of the host genotype in response to Cd and Zn stress **(**Fig. 4b, Supplementary Tables S6, S7**)**. There was a strong overlap between the genes that were differentially regulated by Cd and Zn. The Cd treatment induced expression changes in 1035 genes, out of which 736 (71.1%) genes were also differentially expressed in response to Zn, and in both cases, more genes were downregulated **(**Fig. 4c, d**)**. In the WT, the expression profiles of these genes were very similar in these two treatments, and also similar to the WT vs. mutant in control conditions based on combined cluster analysis of gene expression and plant genotype-treatment **(**Fig. 4c, d**)**, suggesting that both Cd and Zn homeostasis depends on *MtABCG36*. Furthermore, despite being a micronutrient, Zn acts similar to Cd, though the latter does not have a known nutritional role in plants.

**Fig. 5:**
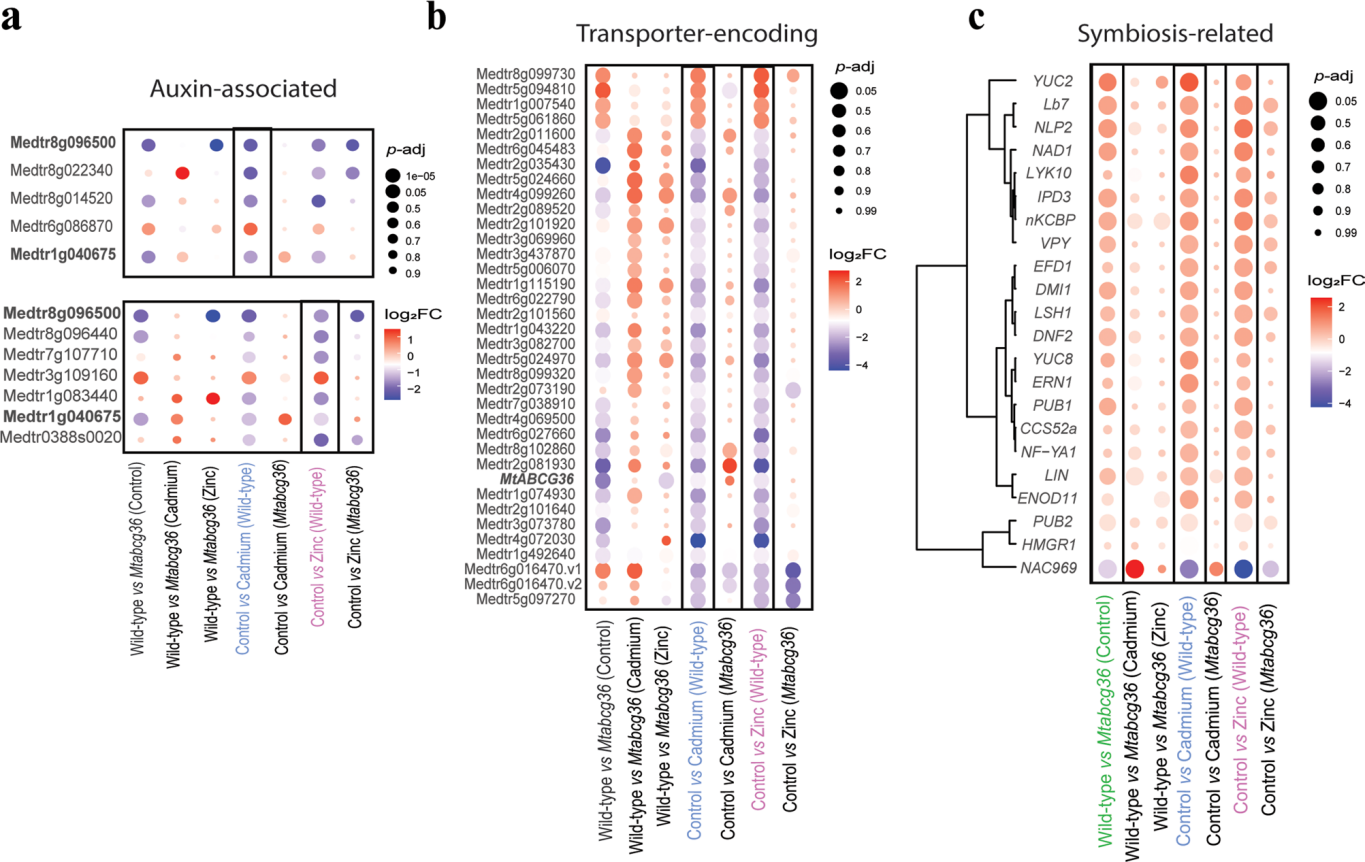
Cadmium or zinc-responsive host plant genes in the nodule associated with auxin, transporters, or symbiosis. (**a**) Dot plots representing auxin-associated DEGs under cadmium (upper panel) and zinc (lower panel) treatments in the nodule. Genes observed in both the Cd and Zn sets are shown in bold. (**b**) Dotplot of DEGs encoding transporters that are observed under both cadmium and zinc treatments. The *MtABCG36* gene in bold is Medtr2g101090 in the *M. truncatula* A17 version 4.0. (**c**) Dotplot of symbiotic genes differentially regulated in the mutant under the control condition, or by Cd or Zn treatment in the wild-type, clustered by log_2_FC. The color of the dots represents the directionality of gene expression. The size of the dots represents the reverse of the adjusted *p*-value; larger dots represent a smaller adjusted *p*-value. The focal groups are enclosed within black boxes and labeled with blue (Cd) and pink (Zn). For a and b, gene IDs on y-axis are Mt4.0 gene annotations, and for c, common names of known symbiosis genes are shown. Gene IDs with v1 and v2 indicate more than one homologs between the R108 Hi-C and Mt4.0 genomes. For all dotplots, the genes may or may not be differentially regulated in combination of genotypes and treatments other than the ones specified. DEGs were defined as: (a, b) |log_2_FC| > 1; *p*-adj < 0.05; and (c) *p*-adj < 0.05.

ABC transporter genes in *M. truncatula* have evolved into a large superfamily comprising several phylogenetic clades^15^. Multiple ABC transporters in the G clade (ABCG) are involved in homeostasis of the plant hormones abscisic acid, auxin, and cytokinin **(**Supplementary Fig. S7**)**^16,17,30,31^. Hence, we explored our transcriptome data for possible connections between *MtABCG36* and hormone homeostasis, focusing on these three hormones. No abscisic acid-associated gene was differentially regulated by Cd or Zn in the WT **(**Supplementary Tables S8, S9**)**. Only one cytokinin-associated gene (Medtr4g044110: putative cytokinin dehydrogenase) was regulated by Zn, and none by Cd in the WT **(**Supplementary Tables S8, S9**)**. In contrast, several genes associated with auxin were differentially regulated by both heavy metal treatments in the WT. Two genes (Medtr1g040675, auxin-induced protein IAA4 and Medtr8g096500, putative small auxin-up RNA) were shared between the Cd and Zn treatments. Most of these genes were not regulated by the treatments in the mutant, suggesting a role of *MtABCG36* in auxin homeostasis in nodules **(**Fig. 5a, Supplementary Tables S8, S9**)**.

Given the overlap between Cd and Zn-responsive host plant DEGs in the WT nodules, we hypothesized that several transporter-encoding genes would be co-regulated by these treatments. Indeed, several genes, including putative ABC transporter genes were co-regulated by Cd and Zn in the WT **(**Fig. 5b**)**. Among the upregulated genes in both Cd and Zn stress conditions, were *MtYSL3* (Medtr1g007540; AT5G53550) and *MtMRP3*, an ortholog of *AtABCC3* (Medtr5g094810; AT3G13080), which have both been shown to be involved in Cd transport or vacuolar sequestration in *A. thaliana*^32,33^. Loss of function mutations in *MtYSL3* have also been shown to affect Fe and Zn uptake and distribution in *M. truncatula* R108 ^34^. Multiple such genes were also differentially regulated in the mutant in the control condition, suggesting once again, a convergence of metal stress response and *MtABCG36-*regulated processes in rhizobium-legume symbiosis. As expected, *MtABCG36* (Medtr2g101090) was significantly downregulated in the mutant compared to WT in the control conditions **(**Fig. 5b, Supplementary Table S10**)**.

*M. truncatula* forms indeterminate nodules, and a single nodule encompasses all the stages of development that occurred during root nodule symbiosis^8^. We focused on well-characterized host symbiotic genes and studied their expression patterns in response to the metal stresses. Because of the longitudinal gradient of differentiation in nodules, we did not set a cut-off for log_2_ fold-change because we sampled entire nodules, and genes with small changes in expression could still be meaningful. We did not see clear developmental stage/zone-wise expression dynamics **(**Fig. 5c**)**, where the dendrogram on the Y-axis does not suggest clustering of symbiosis genes by developmental stage. The DEGs include early signaling/infection-associated genes (e.g.: *HMGR1*,* PUB1*,*2*,* VPY* etc.), as well as those that function much later (e.g.: *NAD1*,* DNF2*,* NAC969*). Moreover, contrary to the whole transcriptome where the majority of DEGs were downregulated in response to both metals, most of the symbiotic genes were upregulated under one or both metal stresses, a trend previously observed under sodium chloride stress **(**Fig. 5c, Fig. S8**)**^35^. Among these genes were *EARLY NODULIN 11 (ENOD11)*,* LYSM RECEPTOR-LIKE KINASE 10/EXOPOLYSACCHARIDE RECEPTOR 3 (LYK10/EPR3)*,* VAPYRIN (VPY)*, and *ETHYLENE RESPONSE FACTOR IN NODULATION 1 (ERN1).* Unlike most other genes discussed earlier, these genes were not differentially expressed in the mutant compared to WT under the control condition. Auxin-synthetic *YUCCA 2* and *8* (*YUC2*,* YUC8*) were upregulated by Cd in the WT but not in the mutant, in line with the differential regulation of several auxin-associated genes discussed above **(**Fig. 5a, c, Supplementary Tables S11-15**)**.

Transcription factors play critical roles in different stages of rhizobium-legume symbiosis^8,36^. Multiple transcription factor-encoding genes were upregulated by both Cd and Zn in the WT nodules but not in the mutant nodules under the same treatments. In addition to *ERN1*, this set included *INTERACTING PROTEIN OF DMI3 (IPD3)*,* NUCLEAR FACTOR YA-1 (NF-YA1)*,* NIN-LIKE PROTEIN 2 (NLP2)*,* ETHYLENE-RESPONSE FACTOR REQUIRED FOR NODULE DIFFERENTIATION 1 (EFD1)*, and, *LIGHT-SENSITIVE SHORT HYPOCOTYL 1 (LSH1).* NLP2 directly induces the expression of *leghemoglobin (Lb)* genes^37^. In our dataset, *Lb1/Lb7* (Medtr5g081000) mirrored the expression profiles of *NLP2* in all conditions and their expression profiles clustered closely, corroborating this transcriptional regulation, and pointing towards a possible interaction between Cd, Zn, and iron (Fe) homeostasis in a host genotype-dependent manner **(**Fig. 5c**).**

The transcription factor-encoding *NAC969* (acronym for *NAM*,* ATAF1/2*,* and CUC2-969*) is induced in senescing nodules and repressed by sodic salt stress in the nodules^38^. In our dataset, this gene was strongly downregulated under both metal treatments in the WT but not mutant nodules. On the other hand, *NAC969* was strongly induced by Cd treatment in the mutant compared to the Cd-treated wild-type. These results suggest complex transcriptional regulation of *NAC969* under metal stresses as a function of the plant genotype **(**Fig. 5c, Supplementary Tables S11-15**)**.

### Heavy metal treatments regulate ion homeostasis in the nodule in a host genotype-dependent manner

**Fig. 6:**
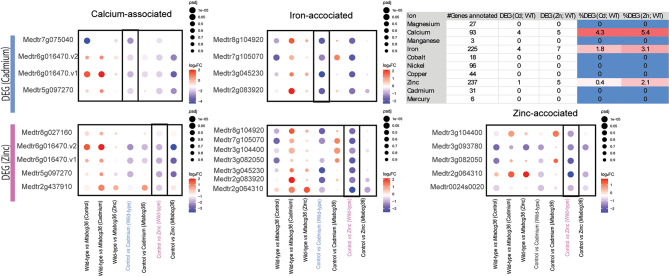
Effect of metal treatments and genotypes on ion homeostasis in the nodule. Dot plots showing plant DEGs that had gene annotations associated with bivalent ions. The upper panels show cadmium-induced DEGs, and lower panels show zinc-induced DEGs in the WT nodule. Gene IDs on the y-axes are Mt4.0 gene annotations and gene IDs with v1 and v2 indicate more than one homolog between the R108 Hi-C and Mt4.0 genomes. Only one zinc-associated gene (Medtr3g467150) was differentially regulated by cadmium. The color of the dots represents the directionality of gene expression. The size of the dots represents the reverse of the adjusted *p*-value; larger dots represent a smaller adjusted *p*-value. Vertical black boxes contain only significant DEGs for those comparisons. For all dotplots, the genes may or may not be differentially regulated in combination of genotypes and treatments other than the ones specified. |log_2_FC| > 1; *p*-adj < 0.05.

Given the role of ABCG transporters in the transport of bivalent metal ions, we searched the set of genes differentially expressed in response to Cd or Zn for associations with these ions based on annotations containing text that includes the following ions: magnesium, (Mg), calcium (Ca), manganese (Mn), iron (Fe), cobalt (Co), nickel (Ni), copper (Cu), zinc (Zn), Cd, or mercury (Hg). No DEG (|log_2_FC| >1, *p-*adj < 0.05) was observed to be associated with Mg, Mn, Co, Ni, Cu, Cd, or Hg. Only one DEG (Medtr3g467150) was found to be associated with Zn in response to Cd **(**Fig. 6, Supplementary Tables S16, 17**)**. Consistent with our Zn treatment, five DEGs with Zn-related functional annotations were observed in the WT. Multiple Ca and Fe associated genes were differentially regulated by Cd and Zn in the WT. No Fe-associated DEGs under any treatment in the WT was differentially expressed in the mutant. These results suggest a role of *MtABCG36* in Fe homeostasis in the nodule, in line with the differential regulation of leghemoglobin genes such as *Lb1/Lb7* in the mutant **(**Figs. 5c and 6, Supplementary Tables S16, 17**)**.

**Fig. 7:**
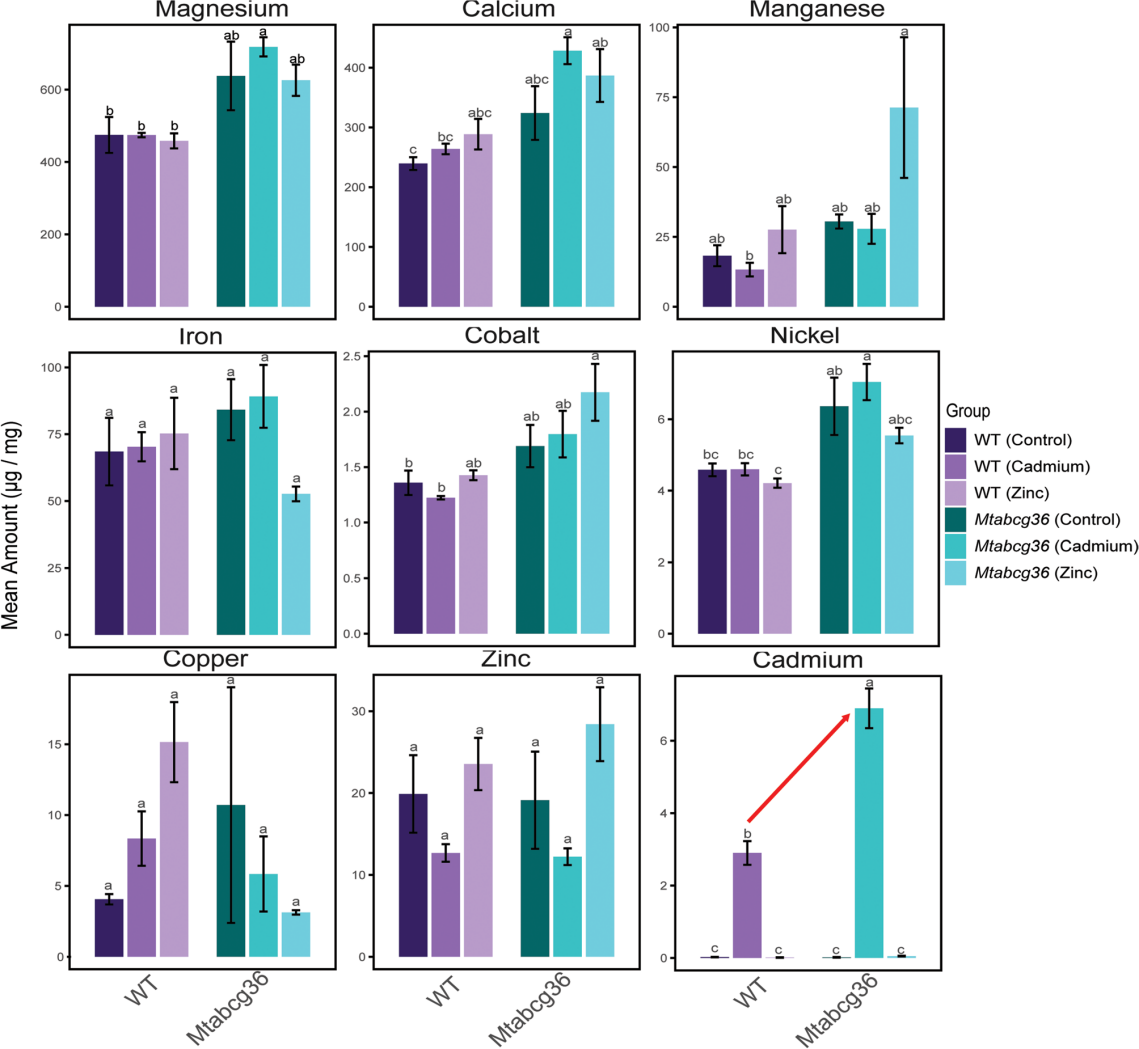
Quantification of elements in the nodules using inductively coupled plasma mass spectrometry (ICP-MS). Bar plots show metal ion accumulation (in microgram per milligram nodule tissue) in the wild-type (WT) and *Mtabcg36* mutant nodules treated with cadmium, zinc or control conditions. Error bars indicate standard error of the mean (SEM). The red arrow in the Cadmium panel highlights the increased accumulation of Cd in the mutant nodules compared to the WT under Cd treatment. Letters display the Tukey’s Honestly Significant Difference (HSD) test for multiple comparisons at α = 0.05. When common letters are shown above any bar, they are not significantly different.

Taking a deeper look into the connection between *MtABCG36* and homeostasis of bivalent ions, we quantified these ions in the WT and mutant nodules in the presence of Cd or Zn, or under the control conditions using inductively coupled plasma mass spectrometry (ICP-MS) **(**Fig. 7**)**. We observed that overall, Mg ion concentration was the highest in nodules amongst all others, congruent with the fact that it is the bivalent cation with the highest concentration in cells^39^. Using tests for multiple comparisons, we did not observe any significant differences in the concentrations of any ion, either between the two genotypes, or in response to Cd and Zn treatments, except Cd. Cd ions showed an expected, significant increase in response to Cd treatment in the WT nodules. This concentration was significantly higher in the mutant under the same condition, suggesting that MtABCG36 could act as a Cd exporter, as previously shown in Arabidopsis **(**Fig. 7**)**^21^.

## Discussion

Leveraging the power of dual transcriptomics to simultaneously compare gene expression dynamics in both symbiotic partners, we observed patterns of responses to these stresses in both partners that were largely contingent on the plant genotype. We harvested nodules for dual transcriptomics under conditions that had clear effects on the shoot and root biomass, as well as nodulation. The Zn treatment resulted in increased shoot biomass, reflecting the nutritional role of Zn in plants, while Cd did not have this effect on shoot biomass. Root biomass and the number of nodules/root biomass were negatively correlated under both metal treatments, as expected due to tradeoffs for resource allocation in root tissues. Thus, corroborating previous findings that nodulation is more sensitive to ionic imbalances compared to general plant growth^35^. Both Cd and Zn increased root biomass and inhibited nodulation, likely reflecting decreased carbon flux towards sustaining symbiosis under a stressed environment^40^. The plant genotype was critical to nodulation, and nodulation was severely impacted in the mutant **(**Fig. 1**)**. Together, we observed a strong effect of both the heavy metal treatments and the plant genotype on nodule development and symbiosis with rhizobia.

### Heavy metal stress response is absorbed by plant cells in nodules

Nodules are comprised of both plant and bacterial cells at various stages of differentiation. These cells possess their own genomic mechanisms for processing nutrients or toxic ion transport. If the plant cells are unable to maintain homeostasis in the root environment during ionic stress, then symbiosis with rhizobia, and nitrogen fixation will be impaired^10^. The PCA of the nodule dual transcriptome data revealed the plant clearly clustered by treatment and genotype, while the bacteria showed very little clustering **(**Fig. 2a, b**)**. We found that the rhizobia inside mutant nodules showed considerable differences in gene expression compared with rhizobia in WT plants after the Zn treatment, but such differences were not observed after Cd treatment. A previous study exposing free-living *S. meliloti* to Cd and Zn stress demonstrated changes in gene expression under both treatments and highlighted a role of exopolysaccharides in Zn, but not Cd stress response^41^. We did not observe any *exo* genes differentially expressed in the mutant nodules under Zn treatment. Furthermore, the genes previously identified in heavy metal tolerant *Mesorhizobium metallidurans* and *S. meliloti* such as *CadA* and other ATPase membrane transporter-encoding genes^42–44^ did not show expression changes in *S. meliloti Sm2011* in the nodules in our study. The differences in these studies raise two primary possibilities: (a) the mechanism of Zn stress tolerance between free-living and symbiotic conditions are different, and/or (b) the host genotype has a role to play, as previously observed^45^. In our study, because we found that very few rhizobial genes responded to the Cd or Zn treatment in both WT and mutant nodules when compared with control conditions, it appears that the host absorbs most of the stress caused by Cd or Zn inside nodules. When comparing the post Zn treatment conditions, the majority of DEGs following Zn treatment in the rhizobia occurred only when the host was a mutant, which suggests that the WT host is better able to absorb more of the stress caused specifically by Zn.

Because a nodule is a symbiotic organ that is comprised of both plant and rhizobia cells, in principle the stress response could equally affect both types of cells at the whole organ level. However, because the rhizobial cells comprise a symbiosome that is surrounded by plant cells, there is a strong potential for the cell types to show very different responses to an external stress. As such, it is not difficult to conceive that these partners will respond to environmental changes differently. Moreover, the nodules provide a unique niche for nitrogen fixation, where the host and symbiont physiologies are substantially different from their non-symbiotic states, which enables them to play their respective roles in this symbiosis, in addition to compartmentalizing stress to maintain homeostasis necessary for symbiosis. In Medicago nodules, the host and the symbiont respond differently to drought stress^46^. Similarly, heat stress causes symbiont-specific transcriptional changes in photosymbiodemes^47^. We find that not only do the host and the microsymbiont respond differently to the metal stresses, where the host shows large numbers of DEGs, and the microsymbiont showed minimal changes in gene expression (control vs. Cd or Zn in the WT), but the host genotype (WT or *Mtabcg36* mutant) and the environment (Cd or Zn treatment) interact to uniquely influence the symbiont. In addition, while large differences in gene expression were observed in the symbiont in WT vs. mutant nodules after Zn treatment, that is not the case for Cd treatment.

The large number of rhizobial DEGs observed between Zn-treated WT and Zn-treated mutant nodules were not observed in response to Zn treatment alone in either genotype, nor between the genotypes without any treatment. This observation suggests that the Zn-genotype interaction is synergistic, and their combined impact is much stronger than their added individual impacts^29^. In the fields of ecology or toxicology, synergy is a contextual phenomenon, and in our case, the overall directionality is negative, as observed by the downregulation of the majority of genes **(**Fig. 3a**)** which we interpret is the result of the combined stress of the *Mtabcg36* mutant and the Zn stress. The combination of the mutation and Zn stress interaction is further supported by significantly lower nodule number in the mutant Zn-treated plants compared with WT while the root biomass in both WT and mutant is high (Fig. 1), due to the resource allocation tradeoff previously mentioned. Therefore, despite the Zn treatment providing a nutrient supplement for plant biomass, in the mutant plants, this comes at the expense of nodulation. These data suggest that the mutant nodules become especially Zn starved due to increased transport to roots and shoots, resulting in a negative impact on the rhizobia in mutant nodules. Cd stress does not have this strong effect on root and shoot biomass, and Cd is not a requirement for normal nodule formation.

### Shared mechanisms of cd and Zn stress responses in the host

Metal ions participate in various cellular functions and act as macro-, or micronutrients. Zn is an essential micronutrient for plants that acts as an enzyme cofactor, participates in hormone synthesis, photosynthesis, and protection against oxidative cellular damage^48^. However, excess Zn can be toxic to plants^49–51^. During root nodule symbiosis, Zn triggers nodule senescence^52^. On the other hand, Cd is not known to play any nutritional role in plants and is phytotoxic even at low concentrations, although natural variation in tolerance to toxic heavy metals does exist in plants, including Medicago^11,12,53,54^. It is widely known that Zn transport mechanisms have been co-opted in plants to transport toxic heavy metals, particularly Cd^55–57^. We observed an increase in root growth and a decrease in the number of nodules per unit root weight under both Cd and Zn treatments **(**Fig. 1**).** Because root nodule symbiosis in *M. truncatula* evolved from the lateral root developmental pathway^58^our results suggest that both Cd and Zn treatments act as environmental cues that shift the balance between root and nodule development. The similarities in root and nodule phenotypes under these treatments were reflected in our nodule RNA-seq data. Over 70% of plant DEGs observed under Cd treatment were also observed under Zn treatment in WT nodules **(**Fig. 4**)**. These genes included several transporter-encoding, metal and auxin-associated genes, suggesting perturbed ion and auxin homeostasis **(**Fig. 5a, b**)**. Given that our experimental set-up comprised of nutrient-replete growth conditions, the overlap of gene expression profiles between Cd and Zn treatments suggests that these treatments affect the plant using several shared mechanisms **(**Fig. 4c, d**)**.

The majority of the host genes commonly regulated by Cd and Zn in the nodule were downregulated by both treatments, which could imply slowing down of basal metabolism and development as previously reported under drought stress **(**Fig. 4c, d**)**^46^. However, most plant genes known to be involved in root nodule symbiosis were upregulated under these treatments in the WT nodules **(**Fig. 5c**)**. Multiple genes such as *ENOD11*,* LYK10/EPR3*,* VPY*, and *ERN1* that were upregulated by the heavy metal salt treatments in our study have been previously shown to be upregulated in rhizobium-inoculated roots under sodic salt treatment compared to untreated, inoculated roots^35^. Such similar results could imply that under these stresses, the nodules do not reach maturity, and the pool of RNA harvested from these tissues contain the markers of early symbiotic genes in abundance compared to the unstressed nodules. These studies used two different genotypes of *M. truncatula*, R108 and A17. R108 is more sensitive to environmental fluctuations^59^and yet similar genes were upregulated in both genotypes. In field conditions, extreme physical environments often constitute multifactorial stress combinations^60^. These studies identified a subset of symbiotic genes that are sensitive to abiotic stresses, in particular, stresses caused by excess salts of sodium and heavy metals. Manipulation of these genes could be useful in engineering environmentally resilient rhizobium-legume symbiosis.

### An ABC protein in metal stress response during trans-kingdom nutritional symbiosis

ABC proteins are present in all domains of life. While they operate using the conserved mechanism of ATP hydrolysis, flexibility in their substrate-binding ability has made them an integral component of various cellular mechanisms, either via direct transport, or through indirect regulation of downstream processes. Their roles range from multidrug resistance in humans to cellular detoxification in plants that is critical to agriculture^61,62^.

While we expected that the mutation in the host plant would result in the disruption of coregulated transport mechanisms, we also found that the microsymbiont is more sensitive to Zn exposure and accumulation when the expression of the host *MtABCG36* gene is compromised, where over 20% of the rhizobial genes were expressed lower in the mutant compared with WT after Zn treatment. Under the same conditions, less than 2% of DEGs were observed in the host **(**Fig. 2d**)**. Moreover, multiple *nod*,* nif*, and, *fix* genes in the rhizobia are downregulated under these conditions, where the host symbiotic genes are either not differentially expressed, or when expressed, they were typically upregulated **(**Fig. 5c, **Supplementary Tables S1**,** S3)**. Technical complications compelled us to base our analyses on a single insertional mutant. Future studies using CRISPR or similar methods will allow us to more specifically identify the role of *MtABCG36* in mediating response to heavy metals during symbiosis. Nonetheless, our current findings clearly suggest that the host genotype influences ion homeostasis in the microsymbiont inside the nodule.

In *Arabidopsis thaliana*, the ABC protein AtPDR8 (ABCG36) acts as an exporter of Cd^2+21^. We also discovered greater accumulation of Cd in the *Mtabcg36* mutant nodules under Cd stress compared to the WT nodules **(**Fig. 7**)**, suggesting a potential role of MtABCG36 as a Cd^2+^ exporter. Moreover, *AtABCG36* acts in Cd stress response through a mechanism that appears to be conserved between *A. thaliana*, rice, and poplar where loss of function mutations or RNAi in the *ABCG36* orthologs decrease Cd tolerance, increase Cd accumulation in root tissues, and reduce root growth^21,22,63^. Using our phenotypic responses as metrics for changes in Cd resilience, we found significantly reduced shoot biomass, root biomass, and nodule number in the Cd treated *Mtabcg36* mutant plants compared with Cd treated WT plants, indicating a reduction in Cd tolerance in the *Mtabcg36* mutant. Because nodules are an extension of root tissue, our results suggest that the role of *MtABCG36* in Cd stress tolerance in nodules likely has a similar function as a Cd^2+^ efflux protein in roots, but also affects rhizobium-legume symbiosis indicated by even poorer nodulation in the mutant than in the control conditions.

**Fig. 8:**
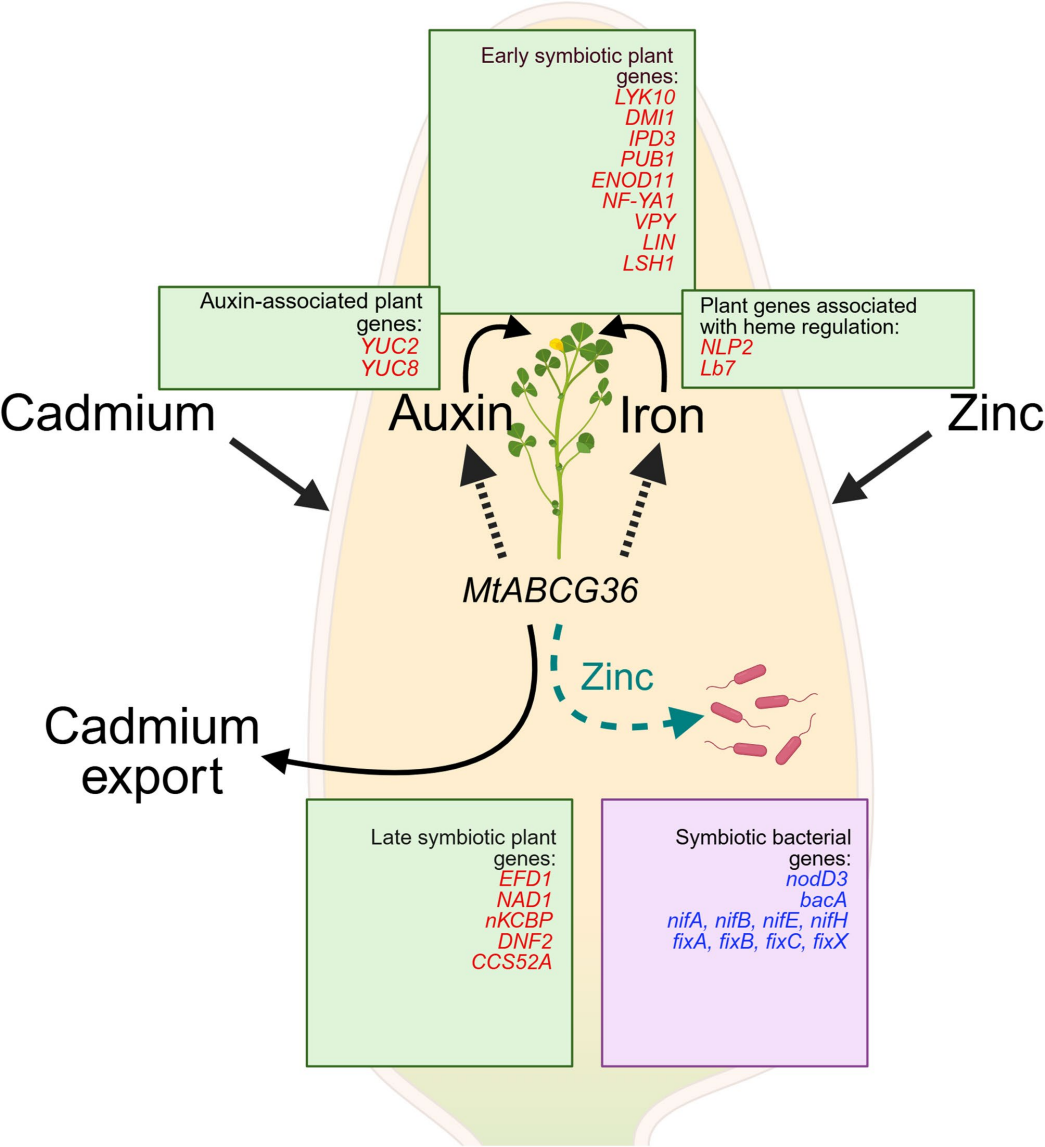
Schematic depicting response of the symbiotic partners to cadmium and zinc treatments as observed by dual transcriptomics and ionomics from nodules. Both Cd and Zn treatments strongly influence the plant transcriptome in the nodule (black arrows). Only Zn treatment shows considerable influence on the bacterial transcriptome in the nodule in a host genotype-dependent manner, when the host plant harbors a mutation in *MtABCG36* (teal arrow). On the plant side, both treatments affect auxin and iron homeostasis, likely mediated by *MtABCG36*. Potential involvement of *MtABCG36* is depicted by dashed arrows. Plant (green boxes) and bacterial (pink box) DEGs are shown. Genes in red and blue fonts are upregulated and downregulated, respectively. The green boxes contain keyenes regulated either by Cd or Zn, or by both treatments. Cd and Zn treatments altered the expressions of the largest sets of plant genes in the wild-type nodules. The complete gene list can be found in Fig. 5c and Table S15. Auxin-associated genes other than auxin biosynthetic *YUC2* and *8* are listed in Fig. 5a and Tables S8 and S9. The pink box shows key bacterial symbiotic DEGs in WT vs. mutant nodules under Zn treatment. This was the only comparison on the bacterial side that showed a considerable number of DEGs. The complete list is in Table S3. MtABCG36 is potentially involved in Cd export, as suggested by our ionomics data and existing literature from other species.

Expression of transporters in the WT in response to both Cd and Zn showed highly similar log_2_FC, significance, and directionality, with mostly downregulation of the transporters. In the *Mtabcg36* genotype under the same two stress treatments, the expression level, directionality and significance changed dramatically **(**Fig. 5b**)**, which showed a shutdown of differential expression of most these transporters. For host plant symbiosis genes, the upregulation of nearly all of the genes **(**Fig. 5c**)** showed parallel expression patterns in all WT conditions, and loss of significant expression changes in all of the *Mtabcg36* conditions. These findings suggest that *MtABCG36* can have strong pleiotropic effects on gene expression affecting nodule development and ion homeostasis, although other genes could be contributing to the observed phenotypes as well. This included two potentially important transporters that are known to regulate Cd^2+^ transport, *MtABCC3* which was strongly upregulated in response to Cd in WT, but downregulated in response to Cd in the *Mtabdg36* mutant, and *MtYSL3* which showed no expression change in the *Mtabcg36* mutant, but was significantly upregulated in response to Cd in WT. Finally, our findings also point towards possible involvement of iron and auxin downstream of *MtABCG36* in heavy metal stress tolerance in nodules^30,64^ **(**Figs. 5a and 6**)**. Because nodule formation is so heavily dependent on iron homeostasis, the combined effects of the *MtABCG36* mutant and Cd stress can affect plant resilience due to poor nitrogen fixation. A schematic highlighting our primary findings is shown in Fig. 8.

We have shown that dual transcriptomics can be a powerful approach for studying plant-microbe interactions by simultaneously analyzing the gene expression profiles of both the plant and the microbe in the context of environmental stress. This approach helped to identify specific genes that are differentially regulated in both the plant and the microbe during symbiotic interactions, and highlighted genes involved in nutrient exchange and symbiotic development. While it appeared that the host plant cells in the nodule tissue buffered much of the heavy metal stress in the *S. meliloti* strain, we nevertheless found important synergistic effects in the rhizobial response to Zn. Further studies using combined ion stresses could be applied to dual transcriptomics to quantify synergistic or antagonistic effects^29,65^ in both symbiotic partners. In addition, most studies that have used loss of function mutations in known ion transporters that result in deleterious effects on nodulation have focused mostly on the phenotypic effects of the host plant^10,34^ with little consideration of rhizobial responses. Therefore, incorporating experimental designs that include genetic variation for tolerance to ion stress in both symbiotic partners, would further advance our understanding of the combined effects of stress tolerance in both partners on the dual transcriptome.

## Methods

### Plant growth conditions and treatments

*Medicago truncatula* R108 was used as the wild-type genotype for all experiments. The *Mtabcg36* mutant is the *Tnt1* insertion line NF2826 previously characterized as *pen3-like*, which was previously shown to have produced fewer nodules compared to wild-type^19^. The annotation for *MtABCG36* is Medtr2g101090 in the *M. truncatula* A17 version 4.0 and MtrunA17_Chr2g0331081 A17 v5.0 reference genome. In the *M. truncatula* R108 Hi-C whole chromosome genome assembly, MtABCG6 is MedtrR108_hic.HiC_scaffold_2.4355. The *A. thaliana* ortholog of *ABCG36/PDR8* is AT1G59870. To germinate seeds, the seeds were scarified for three minutes in sulfuric acid, surface sterilized with 50% bleach and 0.1% tween-20 for three minutes, imbibed for two hours at room temperature, stratified overnight at 4 °C, and germinated in dark at 16/8 h light/dark, at 21 °C and 40% relative humidity.

Germinated seedlings were transplanted to 4” pots containing autoclaved Turface^®^: vermiculite (2:1) with half-strength, nitrogen-free Broughton and Dilworth (B&D) medium^66^ containing necessary macro-, and micronutrients. Zinc concentration in the half-strength medium was 0.25 µM. Four to five days after potting, the plants were inoculated with *Sinorhizobium meliloti* strain *Sm2011* at OD_600_ 0.7-1, and replenished with half-strength B&D medium, supplemented with 0.5mM KNO_3_ as required. We assume this nutrient status to be a nutrient-replete condition. Three weeks post inoculation, the plants were treated with 100µM CdCl_2_ or ZnSO_4_. To the best of our knowledge, no ABC transporter has yet been reported in plants in association with chloride or sulfate transport^61^. The heavy metal stress treatments were based on previous studies^67–69^. Because the goal was to study nodules, the treatments were applied at an advanced stage of symbiosis.

### ICP-MS methods

Nodule samples were collected after one week of stress treatment, i.e., after four weeks of inoculation. To wash off the excess metals from root samples, they were sequentially immersed in 5 mM CaCl_2_, 1 mM MES-KOH, and ultra-pure H_2_O (UPW) for 30 min each. Subsequently, the samples were dried by placing them in a 50 °C oven for a minimum of 48 h. Once the samples were dried and homogenized by crushing, approximately 75 mg was weighed and transferred into tubes for digestion by adding ICP-MS grade 69% HNO_3_ (1017992500, Sigma-Aldrich). They were allowed to sit overnight (to avoid boiling over) and then transferred on a Digiprep heat-block set at 95 °C for 4–5 h. Finally, the samples were diluted 4-fold with UPW and were ready for analysis. We used ICP-MS (NexION 350D Model, Perkin Elmer) to determine the concentrations of metals in the samples. The instrument was calibrated using an environmental standard mix (N9307805, Perkin Elmer), and 89Y and 115In were used as internal standards (M1-ISMS-25, Elemental Scientific). The concentrations were converted from µL/g to µg/g by normalizing to the dry weights of the samples.

### Dual RNA-seq from nodule samples

Total RNA from the nodules were extracted using the Qiagen RNeasy kit and treated with DNase to remove genomic DNA contamination. The samples were then run on a Fragment Analyzer (Agilent) to evaluate RNA integrity. Construction of the dual RNA-seq libraries and sequencing on the Illumina NovaSeq 6000 were performed at the Roy J. Carver Biotechnology Center at the University of Illinois at Urbana-Champaign. The total RNAs were converted into individually barcoded RNAseq libraries with the Universal Plus mRNA-Seq Library Preparation kit from Tecan, using custom probes against *M. truncatula* and *S. meliloti* rRNA designed by Tecan. Libraries were barcoded with Unique Dual Indexes (UDIs) which have been developed to prevent index switching. The adaptor-ligated double-stranded cDNAs were amplified by PCR for 10 cycles. The final libraries were quantified with Qubit (ThermoFisher) and the average cDNA fragment sizes were determined on a Fragment Analyzer. The libraries were diluted to 10nM and further quantified by qPCR on a CFX Connect Real-Time qPCR system (Biorad) for accurate pooling of barcoded libraries and maximization of number of clusters in the flowcell.

### RNA-seq data analysis

Paired end Illumina reads (150 bp) were trimmed using trimmomatic^70^. Before mapping, the *M. truncatula* R108 Hi-C full chromosome reference genome assemblyand annotation (Kaur et al., 2021) was concatenated with the *S. meliloti Sm2011* genome^72^. The *Sm2011* genome was annotated using using RASTtk (https://rast.nmpdr.org)^73^to update previously released gene annotations of this strain, using a larger collection of wild *S. medicae* strains. This included using the RASTtk annotation pipeline and Orthofinder analysis^45^. The concatenated genome was indexed using STAR^74^. Trimmed reads were then mapped to the concatenated *M. truncatula* R108-*Sm2011* reference genome using STAR. EdgeR^75^ was used to quantify weighted trimmed mean of the log expression ratios (trimmed mean of M values (TMM))^76^ as a normalized read count for quantifying and visualizing relative gene expression values in Cd-, or Zn-treated samples compared with control samples. The R108 gene annotations were merged with Mt4.0 gene annotations using Table S8 from^71^to utilize and identify previously reported genes in the *M. truncatula* literature. Subsequently, functional annotation from Phytozome v13 for *M. truncaulta* v4.0 was incorporated with each of the R108 Hi-C gene models. The Phytozome annotation included KOG, KEGG, ENZYME, pathway, and InterPro annotations as well as the best BLAST hit to the *A. thaliana* genome. Statistical analysis of read counts for each gene was done using DESeq2^77^, separately for the R108 plant genome and the *Sm2011* rhizobial genome to avoid normalization of the count data on the concatenated genome. RNA-seq data was analyzed on RStudio (2023.06.2 + 561 “Mountain Hydrangea”). “pheatmap” (https://cran.r-project.org/web/packages/pheatmap/pheatmap.pdf) and “EnhancedVolcano” were used (https://github.com/kevinblighe/EnhancedVolcano) to generate the heatmaps and volcano plots, respectively.

### Statistical analysis

Plant phenotype and ICP-MS data were analyzed using JMP^®^ Pro 17.1.0 (671353).

## Supplementary Information

Below is the link to the electronic supplementary material.

Supplementary Material 1

Supplementary Material 2

## Data Availability

Raw Illumina data is available at the NCBI Bioproject: PRJNA1137930 : Medicago truncatula nodule RNA sequencing under cadmium and zinc stresshttps://dataview.ncbi.nlm.nih.gov/object/PRJNA1137930?reviewer=m8a3a28hn7imlub6an1vaai2m.
