# Supplementary material for: Partners in root nodule symbiosis respond uniquely to heavy metal stresses in a host genotype-dependent manner
Source: Sci Rep. 2025 Sep 29;15:33518. doi: 10.1038/s41598-025-17827-z (PMC12480905; doi:10.1038/s41598-025-17827-z)
Supplement: Supplementary file 2 — Supplementary Material 2 [file 41598_2025_17827_MOESM2_ESM.docx]

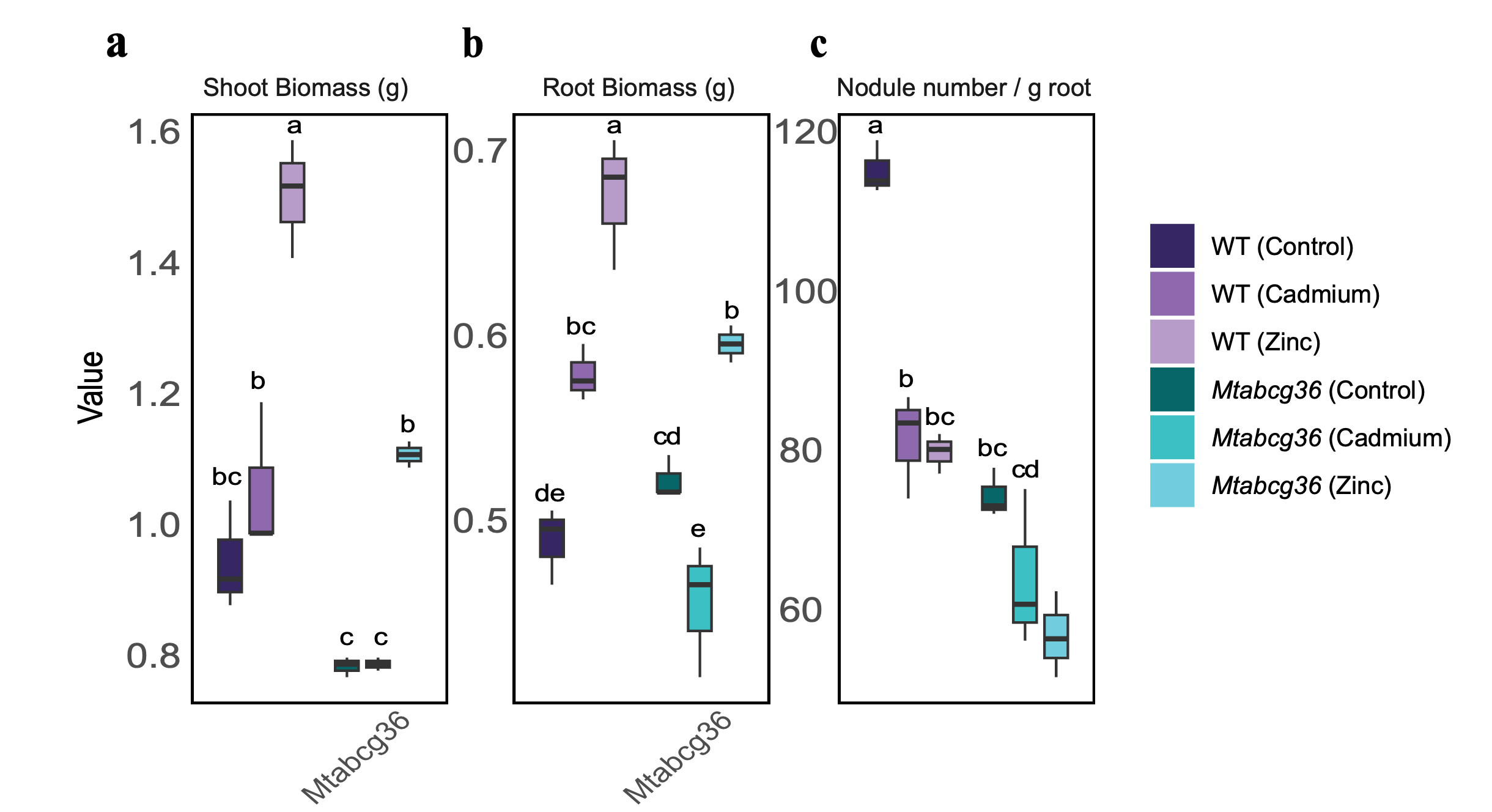


Fig. S1. Quantification of shoot and root biomass, and normalized nodule number, represented as boxplots. Biomass measures for (**a**) shoot, (**b**) root and (**c**) nodule number per gram of root in wild-type (WT) and *Mtabcg36* mutant plants that were inoculated with *Sinorhizobium meliloti Sm2011* and treated with cadmium, zinc, or untreated (control). One week after the treatment, the shoot, root, and, nodules were harvested. Biomass measurements used fresh weight. Five plants were tested in each condition, and the experiment repeated three times. The number of nodules indicates total nodules pooled from five plants. Error bars indicate standard error of the mean (SEM). Letters display the Tukey’s Honestly Significant Difference (HSD) test for multiple comparisons at α = 0.05. When common letters are shown above any bar, they are not significantly different.


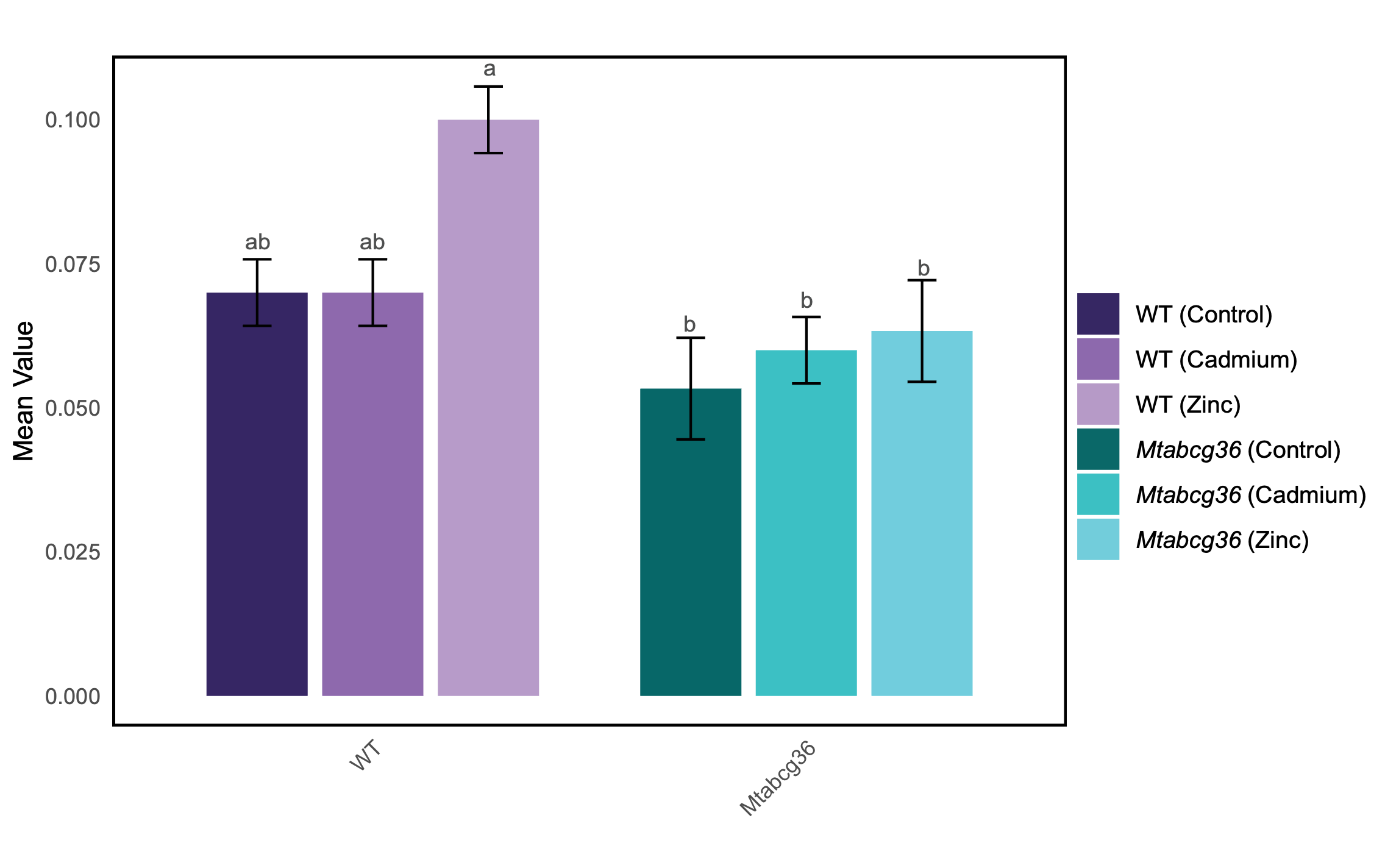


Fig. S2. Quantification of nodule biomass**.**

Nodule biomass measures (g) in wild-type (WT) and *Mtabcg36* mutant plants that were inoculated with *Sinorhizobium meliloti Sm2011* and treated with cadmium, zinc, or untreated (control). One week after the treatment, nodules were harvested. Biomass measurements used fresh weight. Five plants were tested in each condition, and the experiment repeated three times. The number of nodules indicates total nodules pooled from five plants. Error bars indicate standard error of the mean (SEM). Letters display the Tukey’s Honestly Significant Difference (HSD) test for multiple comparisons at α = 0.05. When common letters are shown above any bar, they are not significantly different.


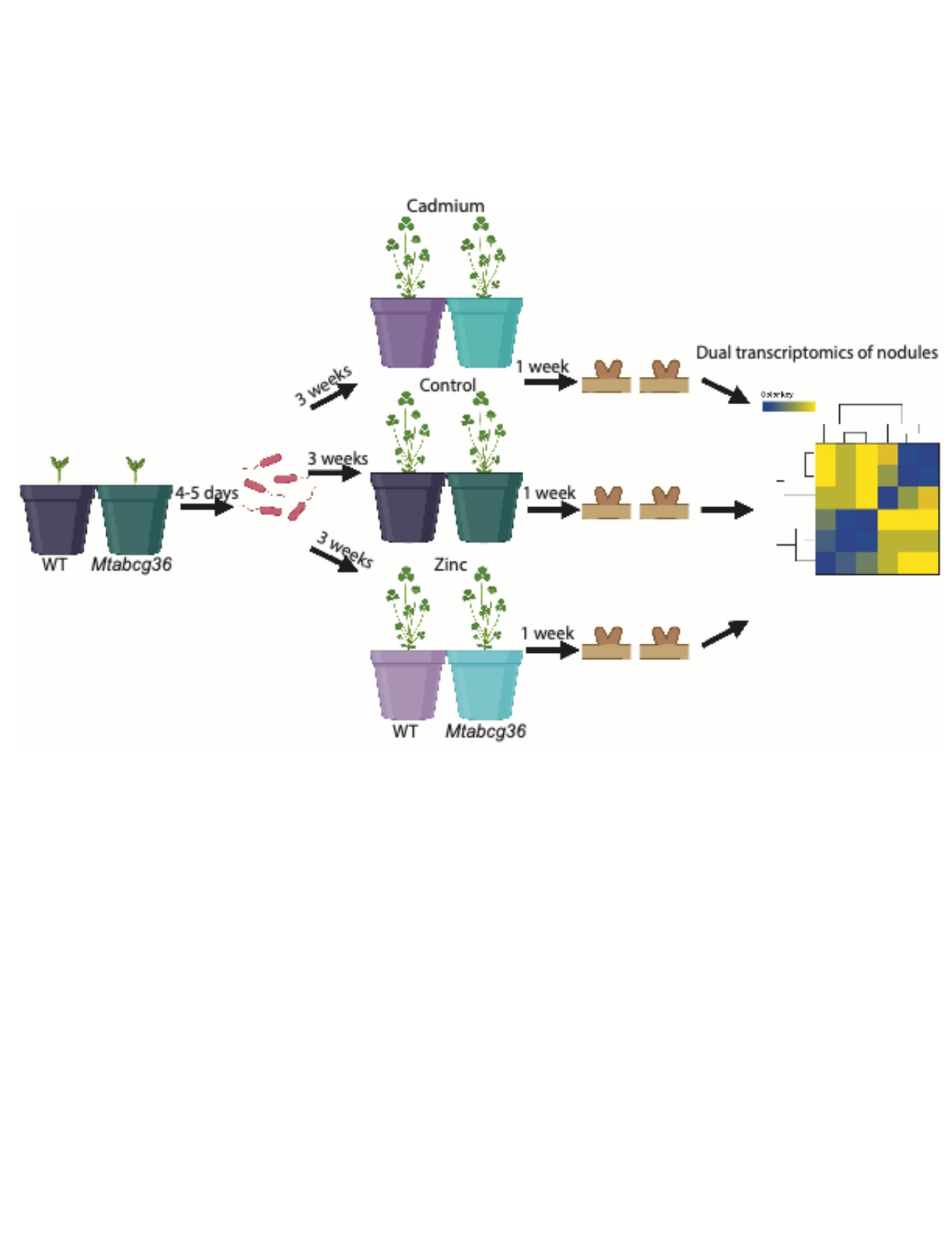


Fig. S3. Experimental design. Wild-type (WT) and *Mtabcg36* mutant plants were inoculated with *Sinorhizobium meliloti Sm2011* four days after potting. Three weeks post-inoculation, both genotypes were exposed Cadmium, Zinc, or Control treatment. One week after the treatment, the nodules were harvested and subjected to dual transcriptomics.

Fig. S4. Assessment of the quality of dual transcriptomics data from the nodules. (**a**) Total number of genes in the plant and the bacterium (**b**) Sum of counts in each symbiotic partner (**c**) Total counts per library in the plant and the bacterial transcriptome (**d**) Total normalized counts per library in the plant and the bacterial transcriptome

.


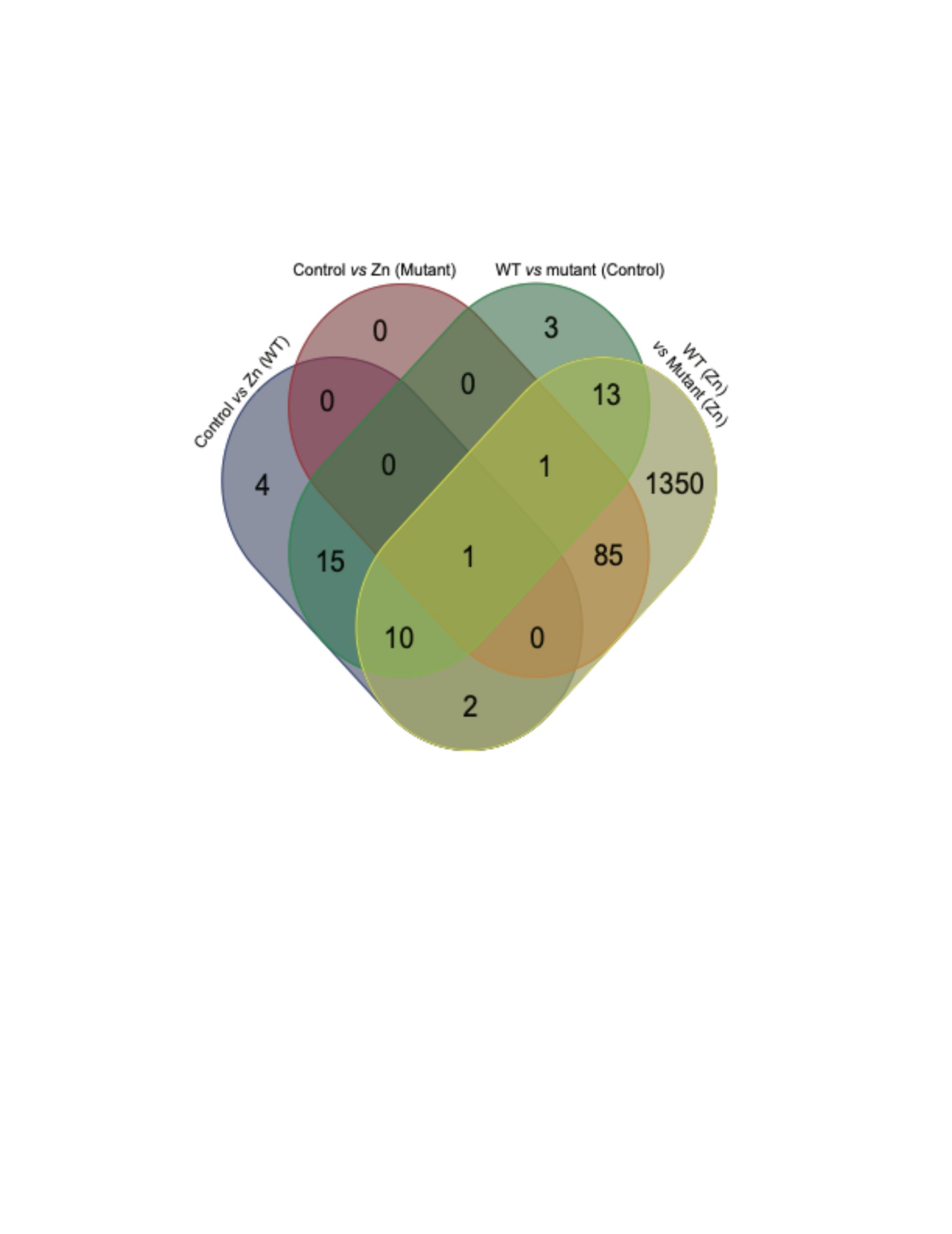


**Fig. S5.** Venn diagram showing rhizobial genes differentially regulated by various combinations of Zn treatment and host genotypes. *p*-adj < 0.05


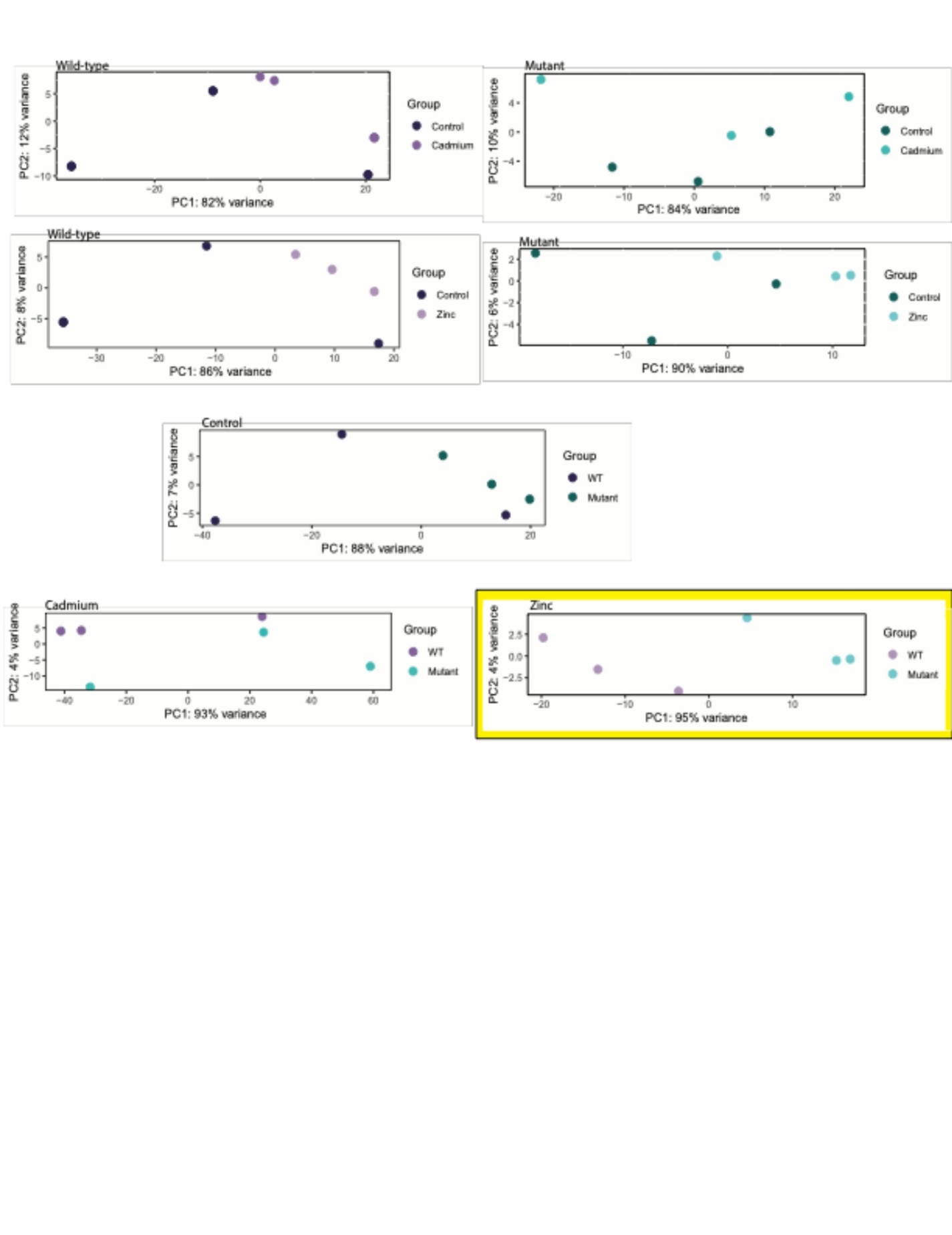


Fig. S6. PCA of all rhizobial pair-wise comparisons shown in Fig. 2B. WT *vs*. mutant under Zn shows the clearest clustering among all the comparisons (highlighted by yellow).


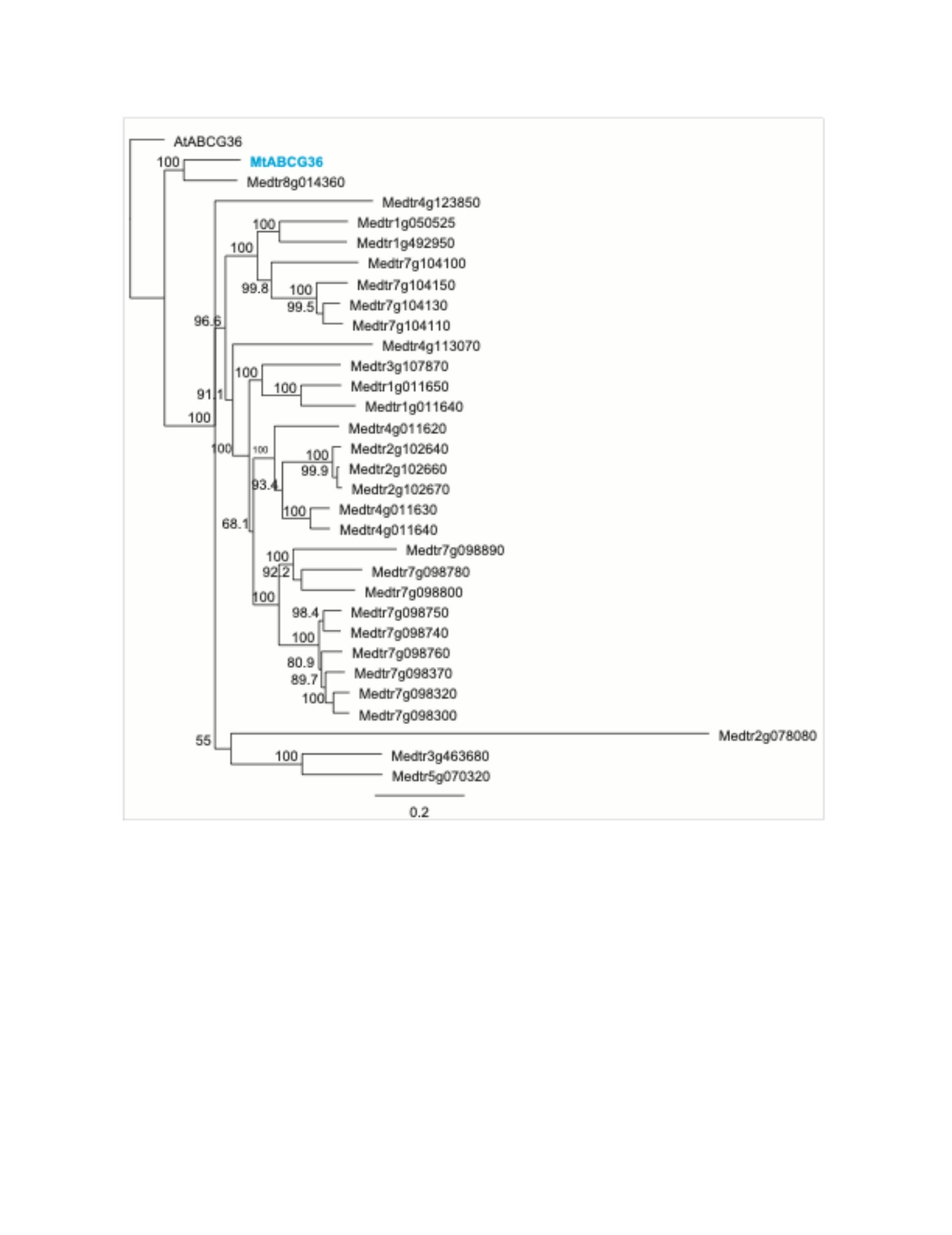


Fig. S7. Phylogeny of full-length *MtABCG*s with *AtABCG36* as the root.

Neighbor-joining tree generated on Geneious. Node label indicates support values in percentage.

Fig. S8. TMM of plant symbiotic genes for Fig. 5C.

Error bars represent standard error of the mean (SEM). The genes are arranged by decreasing order of the range of TMM observed.
